# Effects of Intracellular Force Localization on Cancer Cell Invasion: Revealing Mechanical Trade-offs through Experimentally Validated Computational Models

**DOI:** 10.1021/acsbiomaterials.6c00194

**Published:** 2026-03-08

**Authors:** Amir Shaghoury, Sapir Dadon, Daphne Weihs

**Affiliations:** 1 Faculty of Biomedical Engineering, 26747 Technion − Israel Institute of Technology, Haifa 3200003, Israel; 2 Department of Mathematics and Statistics and the Data Science Institute, Faculty of Science, Hasselt University, Diepenbeek 3590, Belgium

**Keywords:** mechanobiology, cancer cell invasiveness, invasive forces, cytoskeleton, finite element modeling

## Abstract

Metastasis, leading to 90% of cancer-related deaths, is driven by invasive forces exerted by cancer cells on their microenvironment. While actin is central to force generation and motility, the effects of intracellular force-localization during invasion remain largely unexplored. We previously demonstrated, in a clinically relevant assay, invasive cancer cells indenting soft, elastic gels to cell-scale depths, and developed corresponding experimentally validated finite element models. Here, we applied those models to investigate how the force-application location, above (top) or below (bottom) the nucleus, affects invasion efficiency. Under low force-levels (≤100 nN), top-applied forces produce 35–42% deeper indentations than bottom-applied forces, with modest increases in intracellular stress, indicating potentially increased invasiveness. However, with top-applied forces, ∼10% less stress is transmitted to the gel, suggesting less effective microenvironmental mechanical interaction. In contrast, under higher forces (≥150 nN), bottom-applied forces become more effective, transmitting >15% more stress to the gel, with indentation depths becoming comparable between top- and bottom-applied configurations, and significantly (>250%) less nuclear stress generated, thereby supporting invasion. These trends are particularly evident when the cytoplasm is softer than the nucleus, as is typical of (invasive cancer) cells. Thus, top-applied forces may support shallow invasion into soft environments, whereas bottom-applied forces mimicking actin-rich, stiff, leading-edge protrusions, optimize deep, forceful invasion with reduced cell-integrity risk. We demonstrate that intracellular force-localization critically influences the mechanical trade-offs between invasion efficiency and cellular stability, potentially offering targets for antimetastatic strategies.

## Graphical abstract

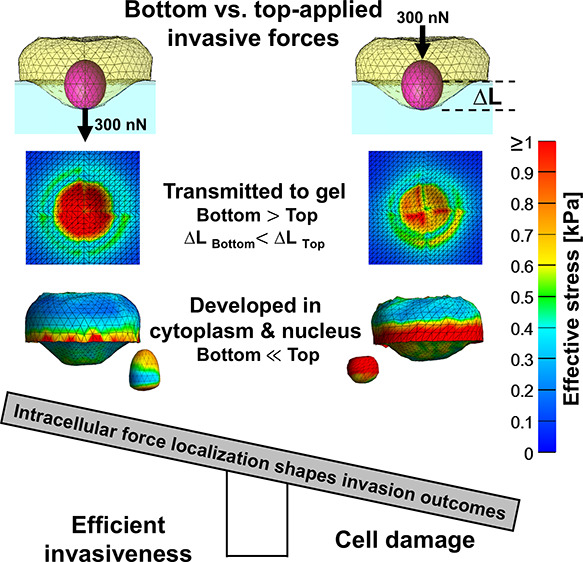

## Introduction

Metastasis remains the primary cause (90%) of cancer-related deaths worldwide, as invasive cancer cells spread from the primary tumor to form secondary tumors at distant body sites. Metastatic cancer cells migrate and invade by changing their internal mechanics and morphology^1–4^ and by applying forces to their microenvironment, particularly to the extracellular matrix (ECM).^5–8^ The mechanics of the ECM significantly influence fundamental cellular behaviors such as spreading, growth, proliferation, migration, and differentiation.^9–11^ Softer cells, despite their reduced stiffness, exhibit greater invasive capabilities through enhanced mechanical adaptability and movement.^1^ This adaptability enables them to deform, migrate, and generate forces, all of which are critical for their invasiveness.^5,7,12,13^ These behaviors are further modulated by the stiffness of the surrounding microenvironment, which not only influences the magnitude of forces that cells generate, but also alters their own stiffness, driving actin remodeling in response to mechanical cues.^3,14,15^ Cell-applied mechanical forces directly support invasiveness,^6,8^ and accordingly, different invasive cancer-cell types have been shown to forcefully push into and indent impenetrable physiological-stiffness gels, thereby providing clinically relevant prognoses for likelihood of metastasis.^16–18^ During the invasive indentation, actin was observed to accumulate at the leading edge of the cell, beneath the nucleus, when deep indentations were attained and its disruption reduced invasiveness.^19,20^ Actin is associated with cell migration and force application by cells,^19–23^ yet no clinical drugs directly target the actin and its machinery, as its role in cell-invasion through dense environments remains unclear. Thus, uncovering how actin localization and related force application promote cancer cell invasiveness may reveal specific cell invasion strategies and offer novel mechanobiological targets.

Revealing how cancer cells exert invasive forces while maintaining their function and structural integrity may uncover novel therapeutic targets. As cells apply force and create an indentation in the gel, the actin cytoskeleton reorganizes and, in deeply indenting cells was shown to concentrate predominantly at the leading edge of the cell, ahead of the nucleus.^19,20,24^ This is unsurprising, as actin plays a key role in cell motility and force application.^19,20,24,25^ The nucleus, which is mechanically linked to the actin cytoskeleton, tended to move into the indentation in the gel, behind the actin;^19,20^ the extent of movement correlated with indentation depth.^19,20^ Specifically, we observed actin concentration beneath the nucleus, at the cell’s leading edge that correlated with deeper indentations, while microtubule localization and distribution showed no significant effect on indentation depth.^19,20,24,25^

In cancer cells, the dynamic cytoskeleton, and specifically actin, enhances invasive capabilities by enabling the formation of protrusions and adaptation to mechanical stresses during metastasis.^19,20^ Actin and the actomyosin machinery have been identified at the leading edge of invasive cells, and, aptly, their disruption reduced invasiveness.^19,20,24,25^ Cytoskeletal disruption, especially of actin, alters cell adhesion, morphology, and force application, directly impacting metastatic potential.^19,20,26–29^ Actin and its machinery facilitate invasive-force application via cell-ECM interactions, e.g., through integrins; those forces allow cells to mechanically deform and invade the surrounding tissue.^19,20,30,31^ The dynamic actin network also ensures that the cells maintain their shape and integrity under external forces or internal mechanical stresses.^32,33^ This is crucial, as significant mechanical deformation can impair nuclear function and compromise DNA integrity.^33–38^ The LINC complex, which links the nuclear envelope to the cytoskeleton, facilitates mechanotransduction of signals from the ECM and cell surface to the nucleus, where gene expression and cellular behavior are regulated.^32,33^ The actin cytoskeleton, in coordination with nonmuscle myosin IIB, is essential for the nuclear deformation and translocation during cell migration. Together with the nuclear lamina, this system facilitates nuclear shape adaptation, mediates mechanotransduction pathways, and provides structural support to protect the nucleus from mechanical stress and damage that may be encountered in confined environments.^39,40^ Interestingly, while actin plays a central role in structure, stability, and invasiveness, no clinical cancer therapies directly target actin.

Here, we directly compare the invasiveness of cells applying forces either from in front or from behind the nucleus, corresponding, respectively, to the leading edge or the rear of the cell, thereby evaluating the role of actin and force localization and in cancer cell invasion. That is, we evaluated the effects of applying forces above or below the nucleus (top- vs bottom-applied forces) on attained cell invasiveness, emulating actin localization patterns observed in shallowly or deeply indenting cancer cells.^19,20^ We have used our experimentally validated, finite element (FE) models^9,24^ of cancer cells applying experimental-scale invasive forces onto a soft physiological-stiffness, impenetrable elastic gel. Those models were modified to include varying cytoplasm stiffness, force magnitudes, and force localization to reflect actin localization in the cell. We then evaluated the effects of force localization (top- vs bottom-applied forces) on the cell-induced indentation depth in the gel and on the total stresses transmitted to the gel as measures of invasiveness. In parallel we assessed the stresses that develop inside the cells, which may affect their structural integrity. Under higher normal forces (>150 nN), the indentation depth of top- and bottom-applied forces demonstrate less than 20% difference for soft, invasive cells (4.4 μm vs 3.7 μm at 300 nN). However, top-applied forces substantially increase intracellular stress, raising nuclear stress by up to 250% relative to bottom-applied forces, while also transmitting less mechanical stress to the gel.In contrast, bottom-applied forces transmit 25% more stress to the gel with lower intracellular stress. This demonstrates that force application beneath the nucleus, at the leading edge of the cell, more efficiently supports mechanical invasiveness while preserving internal structural stability: more stress is transmitted to the gel and less generated inside the cells. The novelty of this study lies in systematically quantifying how intracellular force localization (above vs below the nucleus) alters invasion efficiency and nuclear stress in an experimentally validated FEM framework. To our knowledge, this is the first computational analysis linking actin localization patterns observed in invasive cells to quantitative invasion outcomes.

## Methods

### Cell Model

Cancer cells display roughly circular cross sections with a diameter of 20 μm on soft gels, based on our experiments across various cancer types.^7,13,16,18,19^ Therefore, invasive cells were modeled as three-dimensional, initially hemispherical, with a 20-μm diameter contact area with the gel-substrate, and maintained a rounded overall shape while applying forces to indent the gel.^7,19^ The cells contained a centrally positioned, ellipsoidal nucleus, with average horizontal and vertical diameters of 6 and 8 μm, respectively.^16,19,41^ The cells were modeled as Neo-Hookean materials,^42^ with a Young’s modulus (as the stiffness measure) of 0.8–2 and 2 kPa (see Table 1), respectively, for the cell cytoplasm and nucleus, as nuclei are typically stiffer than the cytoplasm.^43^ The cytoplasm and nucleus were defined with a Poisson ratio of 0.49, as is typical for incompressible cell elements.^44^ Connections between the cell and the underlying gel-substrate and between the cytoplasm and nucleus were implemented using tied facet-on-facet contacts to prevent surface slippage or detachment and to facilitate force transfer. The nucleus was fixed relative to the cytoplasm to ensure synchronized movement during deformation. In addition to the baseline simulations where both the cytoplasm and nucleus were modeled as Neo-Hookean materials, we also implemented a viscoelastic constitutive model for the cytoplasm to better mimic the viscoelastic nature of tissues. The cytoplasm was modeled as viscoelastic represented by G_∞_, G_1_, G_2_ which are 4.05, 34 and 20.2, respectively. Also, the relaxation time, C̅_1_ and C̅_2_ which are 0.58, 5.47 s, respectively.^45^ Here G_∞_ is 4.05 kPa, G_1_ is 34 kPa, G_2_ is 20.2 kPa, and the relaxation time C̅_1_ is 0.58 s, C̅_2_ is 5.47 s, while the nucleus remained Neo-Hookean with a Young’s modulus of 2 kPa, as it is stiffer than cytoplasm and modeled as hyperplastic on several publications.^46–48^ These simulations were used to evaluate whether viscoelasticity influences the relative effects of top- and bottom-applied force localization.

**Table 1:** Mechanics and Physical Scaling of the Modeled Cells and Gels

variable	value or range	source
normal force [nN]	50–350	^12,13,51,56^
cell outer diameter [μm]	20	^19,41^
cell Poisson ratio	0.49	^44^
cytoplasm Young’s modulus [kPa]	0.8–2	^43^
nucleus planar and vertical diameters [μm]	6 and 8	^19,41^
nucleus Young’s modulus [kPa]	2	^43^
cell density [kg/m^3^]	1000	^44^
gel diameter [μm]	450	
gel thickness [μm]	300	^19,41^
gel Young’s modulus [kPa]	2.4	^13,19,41^
gel Poisson ratio	0.48	^50^
gel density [kg/m^3^]	1000	^50^

### Gel Model

An elastic gel model (parameters in Table 1) was used to match the mechanics and physical scaling of the elastic polyacrylamide gels commonly used in our and others’ published experiments.^16,49–51^ The gel-substrate was modeled as an initially flat-surfaced cylinder, with a radius of 225 μm and a height of 300 μm, large enough to approximate a semi-infinite substrate relative to the 20 μm cell size, thereby minimizing boundary effects as well as mechanical interactions with the rigid glass-base used in experiments. The gel was modeled as linearly elastic, consistent with our experiments^13^ and isotropic, with a fixed Poisson ratio of 0.48 based on literature.^52,53^ Our previous dynamic rheological measurements with (Figure S1) showed that polyacrylamide gels respond as linear elastic up to total strains of 80%, well above the effective strains attained in our current simulations. The Young’s modulus of the gels was set to 2.4 kPa to match our experimental invasion conditions,^18,24^ and lies within the physiological range of soft tissues.^13,54^ In all our simulations, the gel was fixed to a rigid substrate,^24^ as in experiments, to prevent gel-detachment or rotation.^7,16^

The gel was modeled as a cylindrical block and the cell as a soft shell-like cytoplasm containing an internal nucleus. We fixed both displacements and rotations on the lateral and bottom faces of the cylinder (all faces except the top face). The top face of the gel was left free so the cell could indent into it. The nucleus rotation was constrained (fixed rotation) to represent anchoring of the nucleus by the perinuclear cytoskeleton and to avoid unrealistically rotations during indentation.

Mechanical interaction between the cell and the gel was defined using a tied facet-to-facet contact between the outer surface of the cytoplasm and the gel top surface. This tied contact enforces continuity of displacements across the interface and therefore approximates strong adhesion (no sliding) between the cell membrane and the gel surface. The nucleus and cytoplasm were also tied facet-to-facet to ensure displacement compatibility at the nucleus-cytoplasm interface and to transmit loads directly into the nucleus.

### Cell Applied Force Application Configurations and Levels

Invasive cells that induce cell-scale gel indentations pull the gel inward and upward at the cell periphery and push the gel downward at the cell center, forming an indentation ‘dimple’ several microns deep (up to 10 μm) beneath the cell body (Figure 1);^7,19,41,55^ the cell nucleus was typically observed at the cells’ leading edge, within the gel indentation-dimple. In our model, a single cell was positioned at the center of the large, initially flat, gel surface, to minimize boundary effects, with push–pull forces applied at the cell perimeter and center (Figure 1, Figure 2). Radially symmetric, in-plane traction forces and normal, upward-pulling forces were applied at the periphery of indenting cells^24,55^ with the total force angled at 50° relative to the gel surface.^55^ The normal force exerted upward at the cell perimeter (and downward at the cell center) ranged between 50 and 300 nN, consistent with experimental results from traction force microscopy and other techniques.^12,13,51,56^

**Figure 1:**
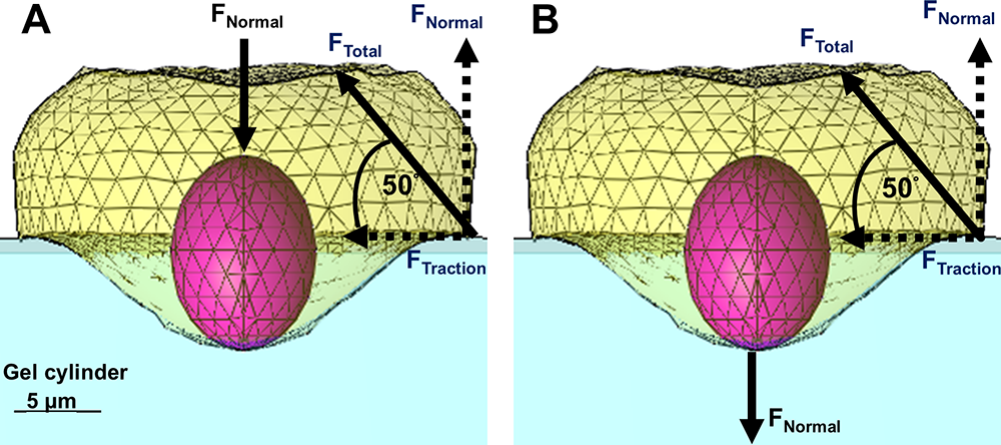
Finite element model of a single cancer cell applying invasive forces to an initially flat, impenetrable, elastic gel-substrate (2.4 kPa stiffness, Poisson ratio of 0.48). Cells apply force uniformly at a 50° around the cell perimeter (*F*_Total_) consisting of a normal pulling force and lateral tractions (i.e., *F*_Normal_, *F*_Traction_) concurrently with normal, downward force (*F*_Normal_) applied either from the top (A) or the bottom (B) of the cell-centered nucleus, being top- and bottom-applied forces; in both configurations the total forces are zero.

**Figure 2:**
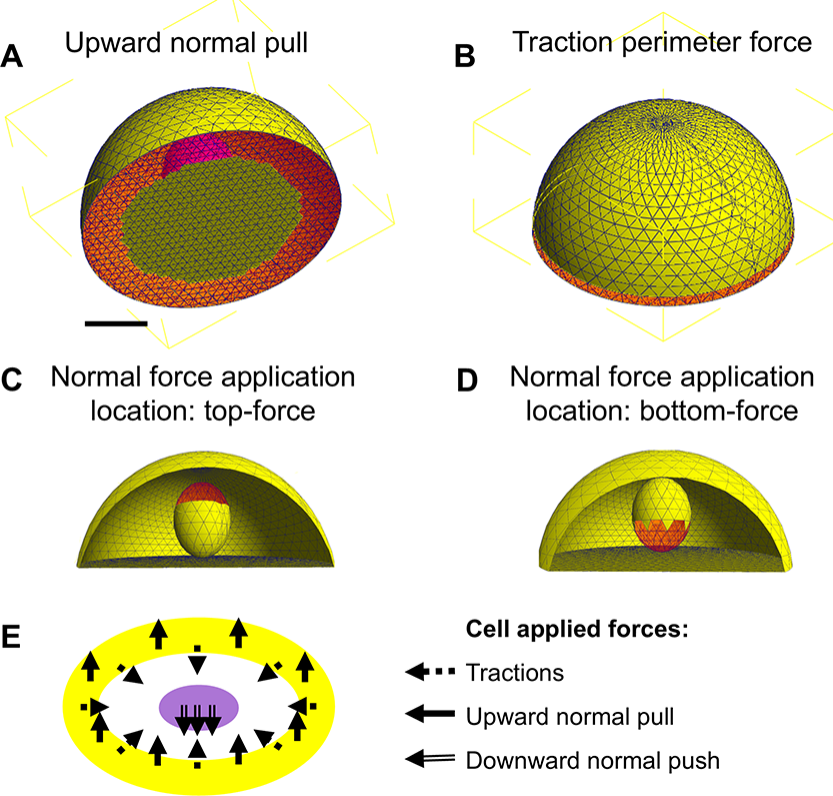
Schematic of force application sites and elements in the finite element model of a single indenting cell. Red-highlighted tetrahedral elements in panels (A–D) indicate regions of force application: (A) Upward normal forces applied at the cell base perimeter. (B) In-plane traction forces applied along the cell perimeter. (C) Downward normal force applied at the top of the nucleus (top-applied force), corresponding to Actin positioned behind the nucleus (rear of the cell). (D) Downward normal force applied at the bottom of the nucleus (bottom-applied force), corresponding to Actin positioned at the leading edge ahead of the nucleus. (E) Schematic overview of the modeled force configuration. Radial traction and upward normal forces (total angle 50°) are applied at the cell perimeter, while a downward normal force is applied either above or below the nucleus.

The traction angle of 50° angle was chosen based on traction force microscopy,^55^ which showed invasive cancer cells exert traction forces at this approximate orientation. We also note that prior sensitivity analyses indicate that small variations (±10°) do not alter qualitative trends. The perimeter forces were applied uniformly along the perimeter at 50° to the gel surface, producing radially symmetric, in-plane traction forces and normal, upward-pulling forces as in our previous works.^9,24^

In the current work, we compared effects on invasiveness when normal forces at the stiff, cell-centered nucleus were applied from above versus below the nucleus (top- vs bottom-applied forces). Specifically, forces were applied via he top or bottom third of the nucleus to the gel through a 6 μm diameter area at the base of the cell cytoplasm (Figure 1, Figure 2). This force localization was designed to emulate the spatial distribution of actin observed in invasive cancer cells, where actin accumulates above the nucleus in shallowly indenting cells, and below it in deeply indenting cells.^19,20^ The volume-portion of the initially ellipsoid-shaped nucleus through which force was applied models the structural effects of dense actin networks adjacent to the nucleus, as observed experimentally.

### Finite Element Models and Analysis

Gel and cell models were meshed using FEBioStudio v1.6.1, simulations were performed using the solver of FEBio v3.5, and results were analyzed and postprocessed in FEBio Studio v1.6.1.^57^ The FE models were solved such that force and moment equilibrium were maintained, i.e., ∇σ_ij_ = 0 in both the gel and the cell; the σ_ij_, is the Cauchy stress tensor, and τ_j_ = σ_ij_ × n_i_ are the applied tractions of force or pressure, where n_i_ denotes the unit vector normal to the gel surface.

The FE models were solved under quasi-static conditions such that both force and moment equilibrium were satisfied.

Force balance in the current configuration is given by Cauchy’s momentum eq 1 or, in index notation (2):

∇×σ+b=0 1

$$a_{\mathrm{ij},j}+b_{i}=0$$ 2

where “σ” is the Cauchy stress tensor and “b” is the body force per unit volume, since gravitational forces on individual cells (∼10–11 N) are several orders of magnitude smaller than the invasive forces applied in our simulations (50–300 nN), body forces were considered negligible and set to zero. Moment equilibrium, in the absence of intrinsic body couples, implies that the stress tensor is symmetric (σij = σji).

Traction boundary conditions were applied according to Cauchy’s traction theorem (3) or, in index notation (4):

t=σ×n 3

$$t_{i}=\sigma_{\mathrm{ij}}n_{j}$$ 4

with “t” the traction vector on a surface with outward unit normal “n”. This formulation ensures that the stresses and applied tractions in both the gel and the cell satisfy the fundamental balance laws of continuum mechanics.

We required convergence of <2% in vertical gel surface deformations (indentation depth),^24^ as well as in stress and strain measures. Final meshes included 637, 7197, and 1260000 linear tetrahedral elements for the nucleus, cytoplasm, and gel, respectively, corresponding to 242, 2013, and 227216 nodes. A meshing convergence test showed that a density of 100 slices was sufficient to maintain accuracy (<2% variation) while balancing computational time-efficiency, thereby ensuring robust and reliable simulation results.

We evaluated the following outcome variables: the cell-induced indentation depth, the total and effective mechanical stresses in the cell cytoplasm and nucleus, and the stress transmitted to the gel. The indentation depth is the maximum deformation of the gel surface caused by the cell, representing the degree of mechanical invasion.^7,16^ The total mechanical stress was computed as the sum of the directional stresses acting in the three-dimensional space^56^ as described in the eq 5:

$$\sigma_{\mathrm{total}}\left(x,y,z\right)=\sum \sigma_{i}\left(x,y,z\right)$$ 5

The effective stress is the von Mises stress. The stress in the gel represents the forces transmitted by the cell.

## Results

We applied our experimentally validated finite element models of invasive cancer cells^9,24^ to evaluate effects of the intracellular force localization on cancer-cell invasion efficiency. Briefly, the cell models consist of an initially hemispherical invasive cancer cell with a centrally positioned nucleus (Figure 1); the Young’s modulus of the cytoplasm and the nucleus were set at 0.8 and 2 kPa, respectively, unless otherwise noted. We simulated two distinct force application configurations: normal forces (50–300 nN) applied either from above or below the nucleus (top- vs bottom-applied forces) onto soft, 2.4 kPa physiological-stiffness gels. We then determined how the location of force application affected the gel indentation depth, stress transmitted to the gel, and the structural stability of the cell, all key indicators of invasive capacity and structural stability.

Top-applied cell forces produce deeper, yet still negligible gel indentations at lower normal force levels (≤100 nN), particularly when the cytoplasm is soft, yet as force magnitude increases, the effect of cytoplasm stiffness inverts (Figure 3). Figure 3A shows that invasive cells achieve, for both top- or bottom-applied forces, micron-scale indentation depths, consistent with experimental results,^16,18,41^ and those increase with the applied normal force. The percentage difference in indentation depth between top- and bottom-applied forces is affected by both force magnitude and cytoplasm stiffness (Figure 3B). Under normal-force of 100 nN and lower, the difference in indentation depth between top- and bottom-applied is approximately 40%, yet as those depths are below 1 μm, the depths and their differences are functionally negligible; indentation depths of <1 μm are well below previously defined depth thresholds for noninvasive cells.^16,19,49^ This matches observations of ∼100 nN adhesive traction forces (do not cause gel indentation) in both cancerous and benign cells on soft substrates.^13^ Differences relating to cytoplasm stiffness are also small in this force range (<5% difference), but this trend reverses at higher forces (≥150 nN).

**Figure 3:**
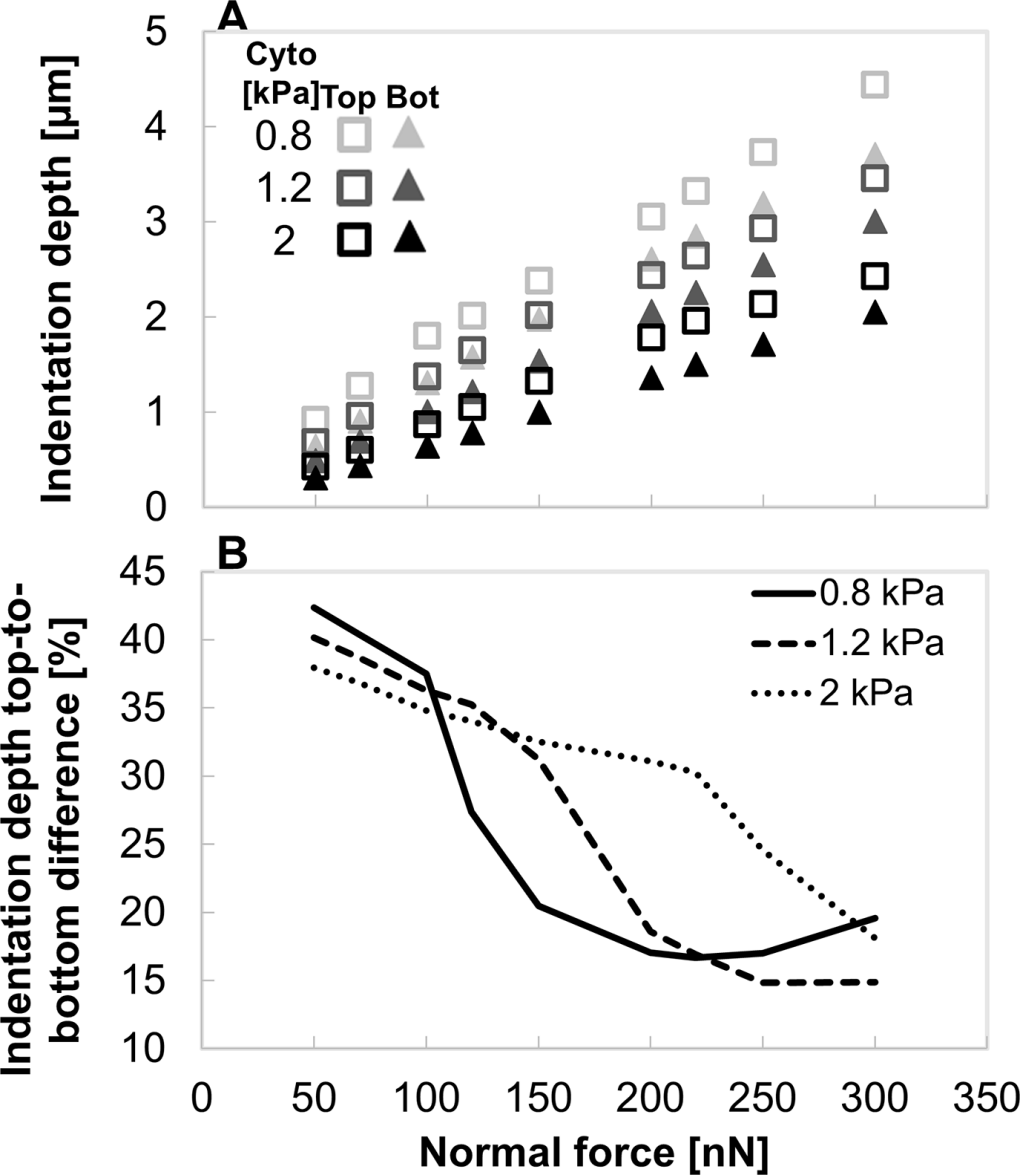
Indentation depth into the 2.4 kPa gel surface as a function of force magnitude, force application location, and cytoplasm stiffness. (A) Indentation increases with force magnitude and is deeper with softer cytoplasm. For all evaluated cytoplasm stiffnesses (0.8, 1.2, and 2.0 kPa), top-applied forces (empty squares) produce greater indentation depths than bottom-applied forces (full triangles); cell nucleus is maintained at 2 kPa stiffness. (B) Percentage difference in indentation depth (top- vs bottom-applied forces) decreases with increasing force with a cytoplasm stiffness-dependent response, reducing to 20% difference at the highest force levels.

In the higher normal-force range (≥150 nN), corresponding to levels observed in different invasive cancer cell types,^9,13,55^ indentations increased nearly linearly, and were deeper for softer cytoplasm; depths match experimental data, e.g., ∼3.9 μm in metastatic pancreatic cells.^9^ The difference in indentation depth between top- and bottom-applied forces declines sharply for soft (0.8 kPa) cytoplasm and more gradually for stiff (2.0 kPa, matching the nucleus) cytoplasm, eventually being under 20% at high forces (Figure 3B). Under increasing forces, soft cytoplasm compresses more easily, reducing the effect of force localization. This agrees with results showing that invasive cells are softer than noninvasive ones,^1,2,35^ and tend to migrate toward softer environments.^58,59^ Soft invasive cells can generate substantial forces to transmit to their microenvironment, yet require intracellular changes.^1,15,35,36^ While top-applied forces produce larger indentation depths on gels, invasiveness efficiency and attainability are better assessed through internal and external stresses, specifically how force localization may impact the cells’ structural integrity and facilitate force transmission to the environment.

Intracellular stresses localize at the periphery and beneath the leading edge at the cell-gel force-contact (Figure 4), with significant differences in both magnitude and distribution between top- and bottom-applied force configurations. At the highest tested force of 300 nN, top-applied forces (Figure 4A) lead to concentrated stresses at the cell periphery and throughout the nucleus, causing it to deform into a rounded shape. Notably, even at low normal forces, the nucleus becomes rounded (Figure S2), unlike the experimentally observed elongated nucleus morphologies, as used at the simulation’s start^7,19^ and may lead to structural damage. In contrast, bottom-applied forces (Figure 4B) generate lower intracellular stresses that are primarily concentrated around the cell periphery and at both the top and bottom of the nucleus; under high forces, the nucleus adopts a pestle-like shape while retaining an ellipsoidal form at lower forces (Figure S2). Importantly, bottom-applied forces result in a significantly higher mechanical stress transmitted to the underlying gel, thereby supporting invasiveness without compromising cell integrity. We note also that the average strain is significantly higher under top-applied forces (Figure S3), and the strain experienced by the cells falls within the range experimentally linked to mechanical damage.^26^ These results suggest that individual cells are more susceptible to damage under top-force application; cells in groups, e.g., during collective migration, may experience reduced effects.^20,60^

**Figure 4:**
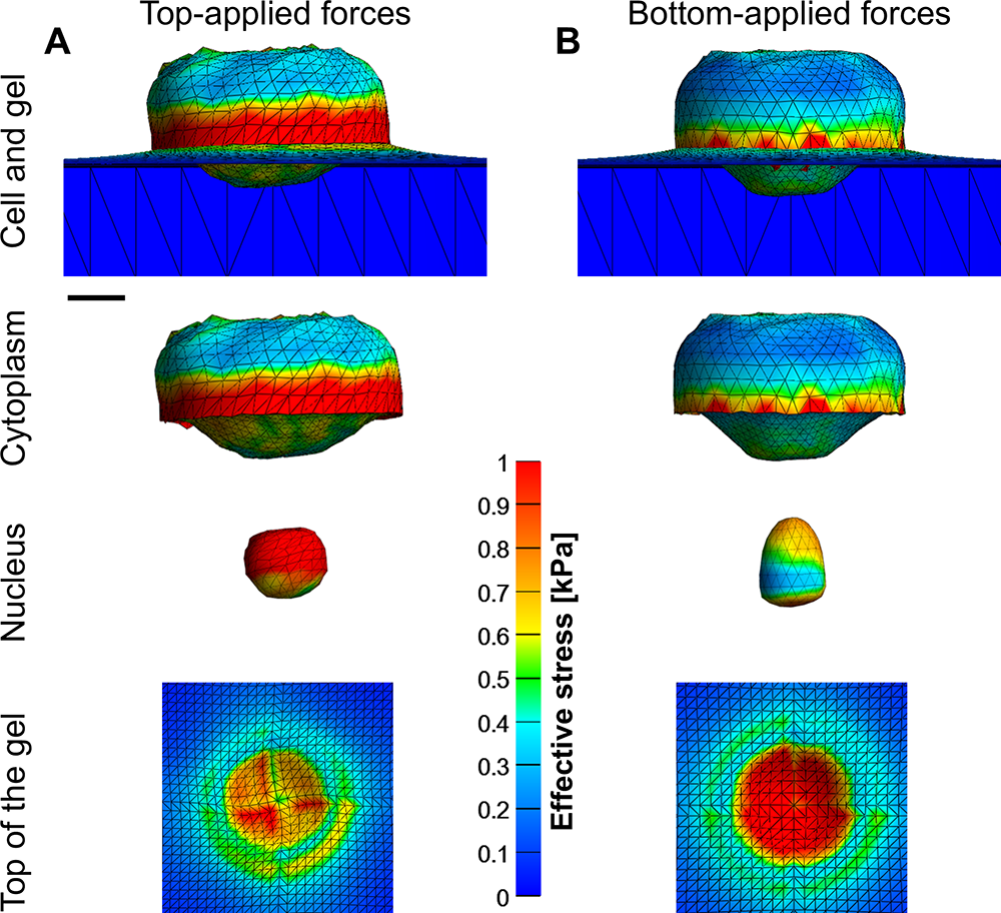
Effective (von Mises) stress distribution in the cell with nucleus (2 kPa stiffness) and cytoplasm (0.8 kPa) for top- vs bottom-applied high-magnitude forces (300 nN) applied on gel ssubstrates. (A) Top-applied forces result in high stress concentrations throughout the nucleus and cell periphery, producing rounded nuclear deformation, with reduced stresses transmitted to the gel. (B) Bottom-applied forces generate lower internal stress and preserve an ellipsoidal nucleus shape, while transmitting more stress to the gel. Scale bar is 5 μm.

Top-applied forces generated higher intracellular stresses in both the nucleus and cytoplasm as compared to bottom-applied forces, with magnitudes increasing with force and depending on cytoplasm stiffness (Figure 5). In the nucleus, bottom-applied forces lead to a gradual, nearly linear stress increase (Figure 5A), consistent with the lower, mode uniform stress distribution seen in Figure 4B. In contrast, top-applied forces produce a sharper rise in stress, particularly above 100 nN, following a potentially bilinear trend^61^ indicating a transition to a higher deformation regime. Under 300 nN of top-applied force, the nucleus stress reaches values that are 250% higher (i.e., 3.5-fold) than with bottom-applied forces (Figure 5B) and are likely to compromise its structural integrity. Similarly, top-applied forces generate greater stresses in the cytoplasm across all force levels (Figure 5C), increasing with force level; a stiffer cytoplasm (2.0 kPa) produced higher overall stress in both force-application configurations under high force levels. Figure 5D demonstrates distinct force-response behaviors depending on cytoplasm stiffness between top- and bottom-applied forces. In stiff cytoplasm (2.0 kPa, matching the nucleus), the stress difference increases gradually and plateaus above 250 nN, indicating a stable, buffered mechanical response. In contrast, a soft cytoplasm (0.8 kPa) exhibits a pronounced peak at 150 nN, followed by a decline, suggesting a force threshold beyond which the cytoplasm structure in the top-applied force may have already undergone a large-scale deformation (as shown in Figure S2). At 300 nN, stress differences converge across stiffnesses, indicating a potential mechanical limit in the system. This convergence likely indicates that under high force-magnitude, the internal stresses - especially under top-applied forces - negatively impact the cell (cytoplasm) structure, regardless of cytoplasm stiffness. Thus, high intracellular stress (>150 nN) may damage the cell mechanostructure, compromise its structural integrity, and reduce its invasive capacity.

**Figure 5:**
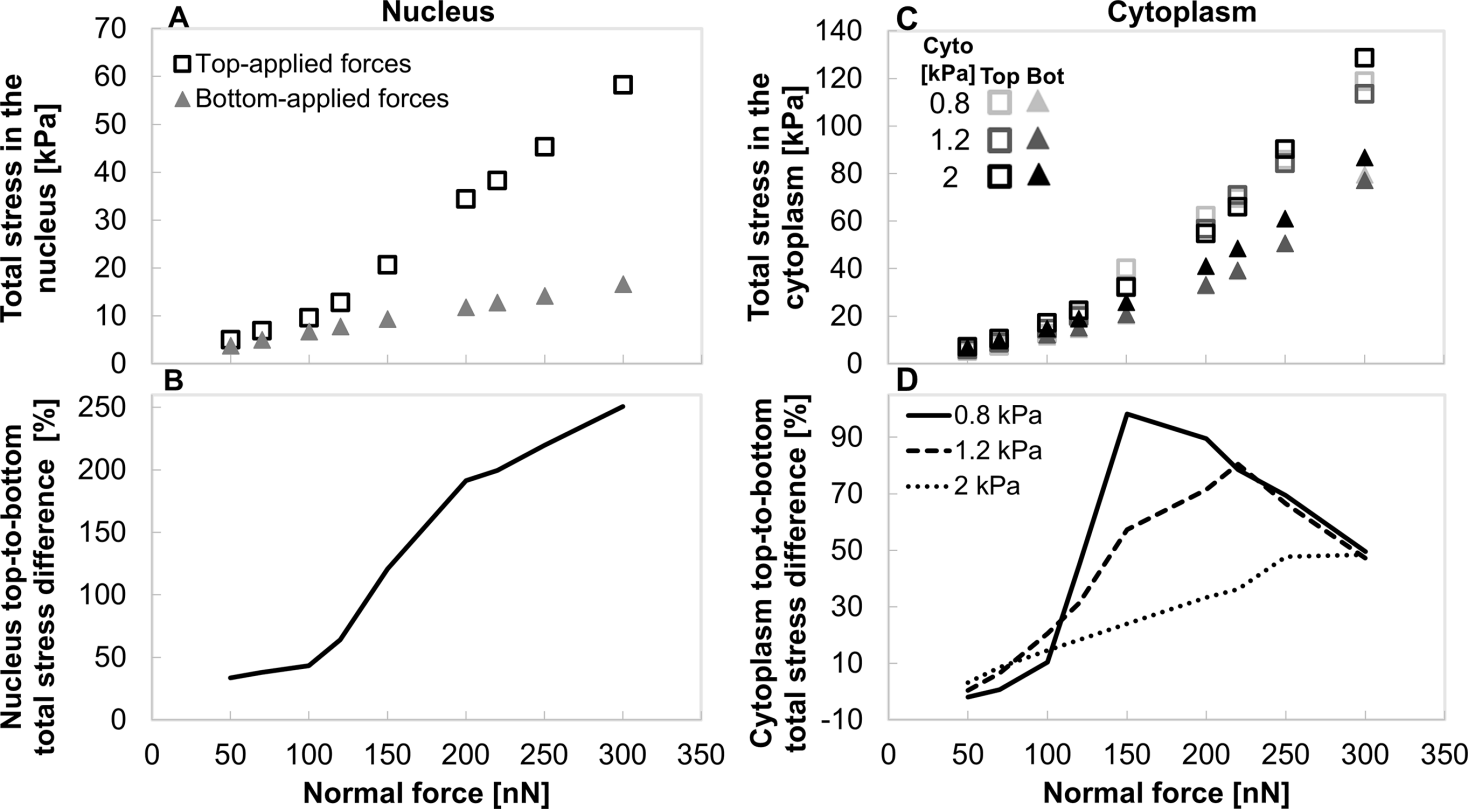
Intracellular stress response in the cell nucleus and cytoplasm under varying force magnitude, force application location, and cytoplasm stiffness. (A) Total stress developing in 2 kPa nucleus (cytoplasm stiffness of 0.8 kPa) under top-applied normal force (empty squares) is higher than with bottom-applied forces (full triangular) and increases more steeply above 100 nN. (B) Percentage difference in nuclear stress between force-application configurations increases with force level, reaching over 250% at 300 nN. (C) Cytoplasm stress increases with force for all evaluated cytoplasm stiffnesses; stiffer cytoplasm typically accumulates higher stress. (D) Percentage difference in cytoplasmic stress varies with stiffness: in stiff cytoplasm (2.0 kPa), differences increase and plateau above 250 nN, while in soft cytoplasm (0.8 kPa), a peak appears at ∼150 nN followed by a decline, likely due to deformation under top-applied forces.

To evaluate the potential effect of cytoplasmic material behavior, we also modeled the cytoplasm as viscoelastic while keeping the nucleus Neo-Hookean. As shown in Figure 7A,B, top-applied forces (300 nN) produced concentrated stresses throughout the nucleus, leading to rounding, whereas bottom-applied forces generated lower, peripheral stresses and preserved an ellipsoidal shape, consistent with elastic simulations (Figure 4). Figure 7C shows that viscoelasticity reduced peak nuclear stresses by ∼42% and decreased the difference between top- and bottom-applied configurations from 250% to ∼175% at high force magnitude (300 nN), reflecting load dissipation and stress relaxation. Indentation results (Figure 7D) revealed that top-applied forces caused deeper gel deformation in both models, with the difference between top to bottom converging to ∼25% at high loads. Additionally, indentation depth increases with applied force and aligns with our previous observations^9,24,49,55^ and many others,^62–64^ so the overall effect on both the cell and the gel remains the same.

Cell invasiveness, driven by applied mechanical force, was evaluated by the stress transmitted to the gel substrate (Figure 6), which contributes to gel indentation (Figure 3); this may also influence invasiveness of nearby cells.^24,29,41^ Figure 6A shows that, for a soft 0.8 kPa cytoplasm, bottom-applied forces consistently transmit more stress to the gel than top-applied forces across all force levels (50–300 nN), with the greatest difference observed at higher forces, consistent with Figure 4. Figure 6B further demonstrates that the percentage difference in stress transmitted to the gel, between top- and bottom-applied forces, varies with force. It remains stable at low forces (50–100 nN), sharply decreases between 100 and 150 nN, and then significantly increases at higher force magnitudes, where the bottom-applied forces become significantly more effective. These trends reflect those in nucleus and cytoplasm stress (Figure 5), further suggesting that top-applied forces become less efficient for invasion beyond 150 nN, likely due to stress concentrating inside the cell instead of being transmitted to the gel. This also ties into the reduced differences in indentation depth (Figure 3). Together, these results indicate that bottom-applied forces are more effective at transmitting mechanical stress to the gel while maintaining lower internal stress-maintaining structural stability and potentially enhancing invasiveness.

**Figure 6:**
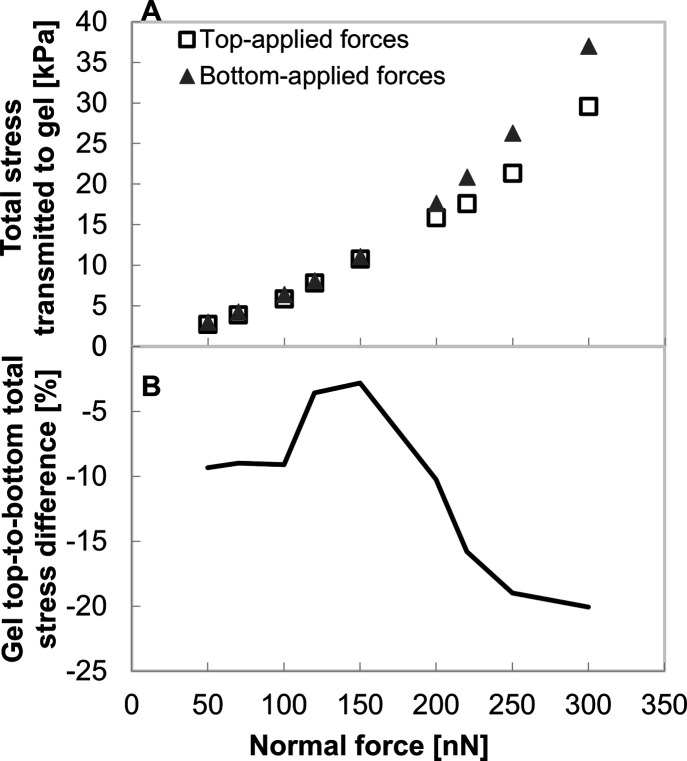
Mechanical stress transmitted to the gel under varying force magnitudes and intracellular force application localization, for cell with nucleus and cytoplasm stiffness of 2 and 0.8 kPa. (A) Bottom-applied forces (full triangles) consistently produce higher gel stress than top-applied (empty squares) across all force levels, resulting in a negative difference, with the difference most pronounced at high forces. (B) The percentage difference in transmitted stress between force configurations is consistently higher (more negative) with bottom-applied forces. It remains steady at low forces (50–100 nN), decreases at 100–150 nN, and increases significantly at higher forces. These trends parallel intracellular stress patterns and suggest reduced invasion efficiency of top-applied forces.

## Discussion

This study demonstrates that the location of intracellular force application: from above or below the stiff nucleus, can significantly influence cancer cell invasiveness, internal stress distribution, and structural stability. Our finite element analysis shows that bottom-applied forces, mimicking actin localization at the leading edge, more efficiently transmit mechanical stress to the extracellular environment while minimizing development of damaging intracellular stress. In contrast, top-applied forces, and especially at higher force magnitudes, lead to high intracellular stress in the cytoplasm and nucleus, risking structural damage while concurrently transmitting less stress to the gel. Our models reflect experimentally observed, actin localization beneath the nucleus in deeply indenting invasive cancer cells,^19,20^ and reveal the quantitative impact and potential biomechanical consequences of the force localization in the cells. We have shown that bottom-applied forces consistently result in greater gel stress transmission and lower nuclear stress, while top-applied forces surpass the previously determined nuclear damage threshold of ∼10 kPa in cancer cells^65^ already in normal forces as low as 100–150 nN (as in Figure 5A), and increasing sharply beyond that point. This suggests a mechanically critical threshold, beyond which top-applied force may compromise nuclear and cellular integrity. Tensile stresses in this range have been shown to induce nuclear membrane rupture^65^ which can lead to nucleo-cytoplasmic leakage^38^ in both normal and cancer cells, and could contribute to DNA damage and genomic instability in the cells.^33–38^ Notably, additional mechanical signatures emerge near this threshold. An additional observation in our simulations was the distinct stress peak occurring in soft cytoplasm (0.8 kPa) under 150 nN applied force (Figure 5D). This phenomenon may reflect the onset of nonlinear cytoskeletal responses at critical loading thresholds. Specifically, actin filaments may undergo buckling or reorganization under compressive forces, leading to transient amplification of cytoplasmic stress before redistribution reduces it at higher forces.^31^ The contribution of this work is the direct comparison of intracellular force-localization modes within an experimentally validated finite element framework, using established constitutive models for simplicity, to reveal how force application above versus below the nucleus influences nuclear stress and invasion efficiency. While explicit nuclear morphometric indices such as aspect ratio or volume change can be extracted from the deformation fields, the present study focuses on force-localization-dependent mechanical trade-offs, using nuclear stress and strain as quantitative mechanical proxies, and leaves detailed morphometric analysis for future extensions of this framework.

Our model refers to both the nucleus and the cytoplasm as Neo-Hookean materials. Modeling the cytoplasm as viscoelastic material^11,43,45^ revealed that time-dependent material behavior reduces absolute stress magnitudes while maintaining the relative mechanical trends observed in the elastic material case. At 300 nN loading, top-applied forces concentrated stresses throughout the nucleus, producing rounding, whereas bottom-applied forces generated lower, peripheral stresses that preserved an ellipsoidal shape (Figure 7A,B), in agreement with the Neo-Hookean simulations (Figure 4A,B). Introducing viscoelasticity reduced peak nuclear stresses by approximately 42% (Figure 7A,B) and narrowed the difference between top- and bottom-applied configurations from 250% to ∼175%, indicating that stress relaxation enables the cytoplasm to dissipate part of the applied load. Despite this attenuation, the overall mechanical behavior remained consistent: bottom-applied forces continued to minimize nuclear stress while transmitting more stress to the gel. Likewise, indentation depth increased with applied force in both models, consistent with our previous findings^9,24,49,55^ and with independent reports showing that greater cellular traction or applied force produces larger substrate deformation.^62–64^ These results demonstrate that cytoplasmic viscoelasticity buffers intracellular stresses without altering the fundamental relationship between applied force magnitude, indentation depth, and the balance between invasion efficiency and structural stability. Additionally, this model does not explicitly simulate actin filament dynamics or cytoskeletal remodeling. Instead, Actin-associated forces were simplified as localized top- or bottom-applied forces, which correspond to experimentally observed actin distributions in shallowly and deeply invading cancer cells.^19,20^ This abstraction captures the first-order mechanical consequences of actin positioning but does not fully reproduce the complexity of actin regulation. Future extensions of the model that explicitly include actin network dynamics could provide more biologically detailed insights into how cytoskeletal remodeling shapes invasiveness.

**Figure 7:**
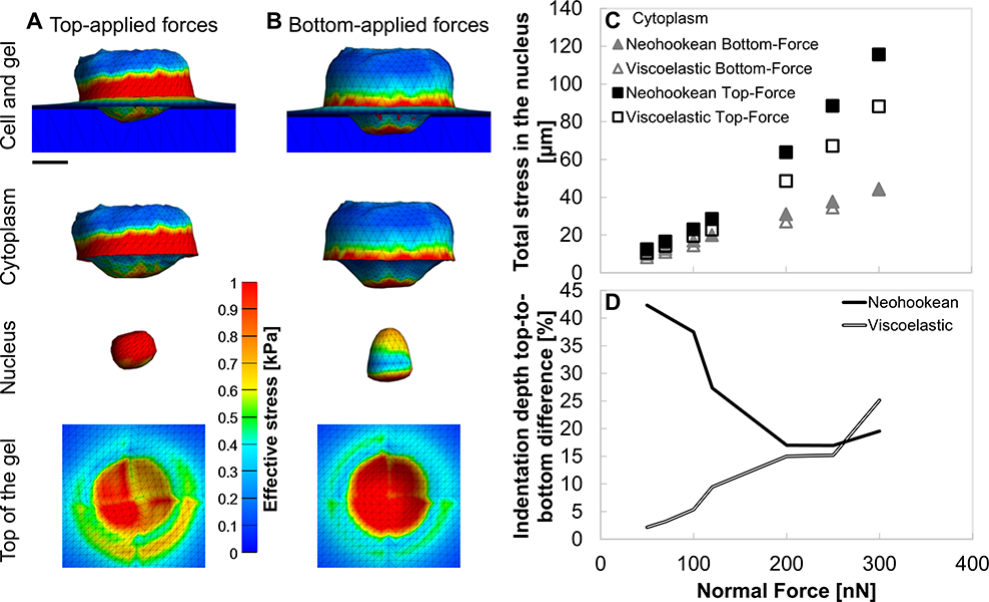
Effect of cytoplasmic viscoelasticity and force localization (for top- vs bottom-applied forces) on nuclear stress distribution and gel indentation (A) Top-applied forces at high magnitude (300 nN) result in high stress concentrations throughout the nucleus and cell periphery, producing rounded nuclear deformation, with reduced stresses transmitted to the gel. Scalebar is 5 μm. (B) In (A, B), colors represent effective stress (0–1 kPa), as indicated by the color scale. Bottom-applied forces at high magnitude (300 nN) generate lower internal stress and preserve an ellipsoidal nucleus shape, while transmitting more stress to the gel. (C) Total stress developing in 2 kPa nucleus (cytoplasm stiffness of 0.8 kPa) under top-applied normal force (empty squares) is higher than with bottom-applied forces (full triangular) and increases more steeply above 100 nN. (D) On Neo-Hookean cytoplasm, the percentage difference in indentation depth (top- vs bottom-applied forces) decreases with increasing force, reducing to 20% difference at the highest force levels. On viscoelastic cytoplasm, the percentage difference in indentation depth (top- vs bottom-applied forces) increases with increasing force with a cytoplasm stiffness-dependent response. However, keeping the percentage of difference close to Neo-Hookean cytoplasm of about 25% difference at the highest force levels.

At low normal-force magnitudes (50–100 nN), especially in cells with soft cytoplasm, top-applied forces produce somewhat deeper gel indentations while maintaining relatively low internal stress. These conditions may support shallow or early stage invasion into very soft tissues, where external resistance is minimal and lower internal stress would remain nondamaging. While in some conditions softened tissues may be encountered *in vivo*,^58^ force levels that lead to indentations <1 μm (as in Figure 3) are typically related to adhesion (and not invasion) of noninvasive/noncancer cells.^19,55,66,67^ However, as force levels would typically increase beyond this range (to ≥ 150 nN), top-applied forces rapidly become inefficient for invasion and structurally hazardous. Cytoplasmic stiffness further modulates these effects. Invasive cancer cells are known to exhibit lower stiffness and greater mechanical pliability than benign or less invasive types,^1–3,15,35,68,69^ enabling migration through dense ECM and confined spaces.^30,31,69^ Our results confirm that softer cytoplasm facilitates deeper indentation and greater force transmission at all force levels (Figure 3), consistent with cytoplasm and nuclear compression observed in invasive cells.^19,30,35,39,69^ In addition, at higher normal-force magnitudes (≥150 nN), the stress difference between top- and bottom-applied forces reduces in soft cytoplasm (Figure 5D) likely resulting from the extensive deformation already sustained by the cells under top-applied forces. While increased cell stiffness can prevent structural damage to cells,^26^ enabling them to withstand external forces more effectively,^36,70^ we show here that this would result in reduced invasiveness, due in part to the structural inflexibility of the cells.^30^

Our results offer a mechanistic basis for experimental observations showing that disrupting actin or cytoskeletal integrity directly reduces invasive capacity of cells.^19,20,25,29^ The actin cytoskeleton connects the plasma membrane to the nucleus through the LINC complex,^32,40^ supporting mechanotransduction and protecting nuclear structure under mechanical stress.^34,35^ Actomyosin-driven forces, primarily concentrated at the cell’s leading edge,^39,71^ are essential for forward protrusions and focal adhesions, and have similarly been observed at the leading edge of forcefully indenting cancer cells.^19,20^ Our findings, therefore, support the premise that accumulation of actin for invasive force application at the leading edge, and ahead of the nucleus, promotes deeper invasive indentations and efficient stress transmission to the gel-substrate, while avoiding potential intracellular structural damage. Even under potentially damaging force conditions, cancer cells may tolerate or adapt through collective behaviors. Top-applied forces may cause mechanical failure in individual cells, yet as cells often invade cooperatively,^24,41^ neighboring cells can exploit already remodeled environments. Additionally, we and others have shown that cytoskeletal, chemotherapy-treated cancer cells release more extracellular vesicles, which contribute to ECM remodeling and niche conditioning at distant sites.^58,59,72^ This plasticity in mechanical behavior and signaling highlights the adaptive strategies used by metastatic cells to overcome mechanical barriers.

Therapeutically, actin remains a challenging target. While the clinical cancer drug Taxol disrupts microtubule dynamics to inhibit mitosis, and can also reduce invasion,^29^ no approved clinical therapies directly inhibit actin. However, the localization and organization of actin, especially at the leading cell edge, beneath the nucleus, may present an indirect strategy for targeting invasiveness without broadly impairing cell viability; the effects of drugs on neighboring noncancer cells are a concern. Interfering with the mechanical coupling between the cytoskeleton and nucleus could reduce nuclear protection during invasion, limiting metastatic efficiency. Our modeling framework directly simulates subcellular force localization and its mechanical consequences, offering a computational approach to probe mechanobiological processes at single-cell resolution, complementing experimental data and highlighting mechanical vulnerabilities in invasive cells. The model, as the experimental system that it is based on, captures physiologically relevant features of invasiveness, including the soft-tissue mechanical environment, such as brain and liver that typically range 0.3–2.9 kPa.^14^ The current model emulates the elastic gel experimental system,^16^ yet it could be extended to include viscoelasticity, dynamic cytoskeletal and ECM remodeling, and interactions with heterogeneous ECMs, to delve into intricacies of the *in vivo* environment. In vivo, cells are subjected to forces that vary not only in magnitude and localization but also in rate. Recent studies show that loading rate itself is sensed by cells, regulating processes such as focal adhesion reinforcement, mechanosensing, and cytoskeletal organization. For example, increasing force-loading rates have been shown to induce a biphasic response in adhesion and YAP localization, with intermediate rates enhancing reinforcement and high rates leading to cytoskeletal softening and fluidization.^73^ Likewise, strain-rate dependent experiments at cell–cell junctions revealed that higher rates elevate stress peaks and promote bond relaxation or failure.^74^ At the molecular scale, integrin-talin-vinculin clutch dynamics are strongly dependent on loading rate, with rapid loading producing behaviors distinct from slow loading.^75^ Together, these findings suggest that incorporating explicit rate-dependent loading into future models could provide deeper insight into how dynamic mechanical inputs shape nuclear stress, deformation, and the trade-offs between invasion efficiency and structural protection.

In summary, this study identifies intracellular force localization as a key determinant of invasion efficiency and mechanical vulnerability. Bottom-applied forces, reflecting actin-rich leading-edge cell protrusions, balances forcefulness with mechanical stability, by supporting invasiveness and effective force transmission to the microenvironment while reducing development of high intracellular stresses. These results highlight a mechanical strategy that cancer cells may use to optimize invasion and suggest new directions for mechano-targeted interventions in metastasis. By revealing how intracellular force localization governs the balance between cell invasion efficiency and mechanical vulnerability, this work provides a mechanobiological framework for understanding how cancer cells optimize invasive strategies while preserving cellular integrity.

## Supplementary Material



## Data Availability

The data that support the findings of this study are available from the corresponding author upon reasonable request.

## Notes

Gel rheology characterization (Figure S1); nuclear morphology and deformation patterns under varying force localization (Figure S2); intracellular strain distributions for top- and bottom-applied forces (Figure S3) (PDF)
