# Supplementary material for: Effects of Intracellular Force Localization on Cancer Cell Invasion: Revealing Mechanical Trade-offs through Experimentally Validated Computational Models
Source: ACS Biomater Sci Eng. 2026 Mar 8;12(4):2457–66. doi: 10.1021/acsbiomaterials.6c00194 (PMC13080771; doi:10.1021/acsbiomaterials.6c00194)
Supplement: Supplementary file 1 [file ab6c00194_si_001.pdf]

**Supplementary materials**

# Effects of intracellular force localization on cancer cell invasion: Revealing mechanical trade-offs through experimentally validated computational models

Amir Shaghoury,[1] Sapir Dadon,[1] and Daphne Weihs[1,2,*]

[1] *Faculty of Biomedical Engineering, Technion – Israel Institute of Technology, Haifa 3200003, Israel*

[2] *Department of Mathematics and Statistics and the Data Science Institute, Faculty of Science, Hasselt University, 3590 Diepenbeek, Belgium*

**Corresponding Author:** Prof. D. Weihs, Faculty of Biomedical Engineering, Technion - Israel Institute of Technology, Haifa 3200003, Israel. Tel. (972) 4-8294134, Fax. (972) 4-8294599, E-mail: daphnew@technion.ac.il

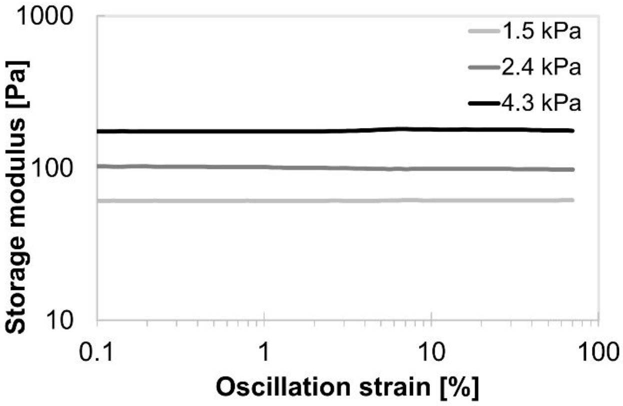


**Figure S1.** Dynamic oscillatory rheology of polyacrylamide gels. The storage (elastic) modulus, G′, of polyacrylamide gels with stiffnesses of 1.5, 2.4, and 4.3 kPa remains constant across oscillatory shear strains up to 70%, indicating linear elastic behavior within this range.

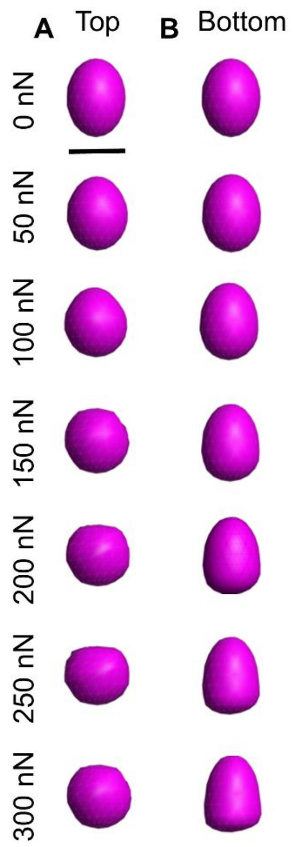


**Figure S2.** Nuclear shape deformation as a function of applied force and its localization. Simulation of a 2.0 kPa nucleus under increasing top- or bottom-applied force magnitudes (0-300 nN). At low forces, the nucleus retains its ellipsoidal shape. At higher magnitudes, top-applied forces cause deformation and rounding of the nucleus, while bottom-applied forces maintain elongated, pestle-like nucleus shape. Scalebar is 6 μm.

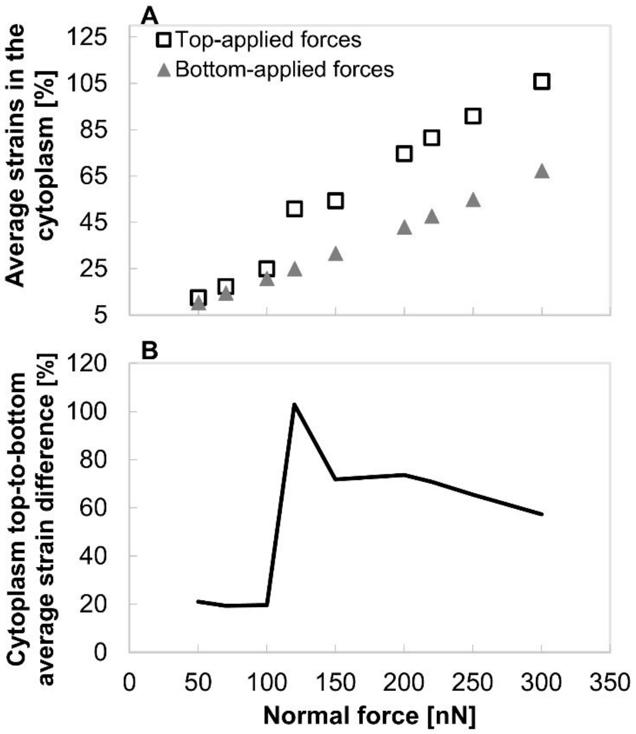


**Figure S3.** Average strain developing in 0.8 kPa cytoplasm under varying force magnitude and force application location. (A) Average strain is consistently higher under top-applied normal forces (empty squares) than bottom-applied forces (full triangles) across all force levels. (B) The percentage difference in cytoplasmic strain remains steady at ~20% between 50-150 nN and is higher (~60%) at higher forces, indicating greater deformation with top-applied forces.
